# New Keypoint Matching Method Using Local Convolutional Features for Power Transmission Line Icing Monitoring

**DOI:** 10.3390/s18030698

**Published:** 2018-02-26

**Authors:** Qiangliang Guo, Jin Xiao, Xiaoguang Hu

**Affiliations:** State Key Laboratory of Virtual Reality Technology and Systems, School of Automation Science and Electrical Engineering, Beihang University, Beijing 100191, China; guoqiangliang@buaa.edu.cn (Q.G.); xiaoguang@buaa.edu.cn (X.H.)

**Keywords:** power transmission line icing, keypoint matching, convolutional neural network, feature fusion, location constraint

## Abstract

Power transmission line icing (PTLI) problems, which cause tremendous damage to the power grids, has drawn much attention. Existing three-dimensional measurement methods based on binocular stereo vision was recently introduced to measure the ice thickness in PTLI, but failed to meet requirements of practical applications due to inefficient keypoint matching in the complex PTLI scene. In this paper, a new keypoint matching method is proposed based on the local multi-layer convolutional neural network (CNN) features, termed Local Convolutional Features (LCFs). LCFs are deployed to extract more discriminative features than the conventional CNNs. Particularly in LCFs, a multi-layer features fusion scheme is exploited to boost the matching performance. Together with a location constraint method, the correspondence of neighboring keypoints is further refined. Our approach achieves 1.5%, 5.3%, 13.1%, 27.3% improvement in the average matching precision compared with SIFT, SURF, ORB and MatchNet on the public Middlebury dataset, and the measurement accuracy of ice thickness can reach 90.9% compared with manual measurement on the collected PTLI dataset.

## 1. Introduction

The development of the smart grid makes higher demands on power transmission line design, operation, and maintenance. However, power transmission lines are vulnerable to icing under the condition of low temperatures, high air humidity and snow. Indeed, the ice load, wind load and dynamic oscillation may all cause a massive power system failure, such as the cable rupture, tower failure as well as transmission line galloping [1,2,3,4]. Consequently, an effective power transmission line icing (PTLI) monitoring and predictive alarm system is critical to ensure power grid safety. 

To this end, some traditional PTLI monitoring methods have been widely used, such as artificial inspection [1], installing pressure sensors [2], building meteorological models [3,4] and so on. In recent years, computer vision-based PTLI monitoring methods have become a new research direction in which icing monitoring can be visualized, convenient and economical [5,6,7,8,9,10]. Some algorithms based on 2D measurement are presented to get accurate ice edges, such as adaptive threshold segmentation [5], edge extraction [6] and wavelet analysis [7]. Then the ice thickness can be calculated through the ratio of pixel widths between edges in normal and icing situations. However, these algorithms have poor performance under complex context or low visibility conditions. Moreover, this 2D estimation method cannot obtain the comprehensive information of icing. To address this issue, methods based on 3D measurement have been introduced to monitor PTLI in order to obtain more accurate information of icing. In [8,9,10], the binocular stereo vision methods were presented to measure ice thickness. The main implementation steps of these methods can be summarized as: camera calibration, keypoints matching, and ice thickness calculation. The accuracy of keypoint matching has a crucial impact on measurement results. Nevertheless, instead of proposing a new algorithm, the aforementioned literature employs the improved classic feature description and matching methods to verify the feasibility of 3D measurement. 

A typical keypoint matching method mainly includes feature description, feature matching and outlier removal. Although the advanced feature-matching methods and outlier removal approaches can effectively enhance the final performance of keypoint matching [11,12,13,14], discriminative feature description is the foundation of the aforementioned processes, especially in the complex PTLI scene. Thus, the focus of this work is on extracting discriminative features and applying it to keypoint matching in the PTLI.

The image noise, similarity of foreground and background, high texture repetition, and low distinction of icing types are the main factors affecting the accuracy of keypoint matching in a PTLI scene. Under such conditions, it is difficult to achieve discriminative features using traditional hand-crafted features, such as SIFT (Scale-Invariant Feature Transform) [15], SURF (Speeded Up Robust Features) [16] and ORB (Oriented FAST and Rotated BRIEF) [17]. As a result, false matching may be caused. In contrast, the features of CNN have certain invariance on translation, distortion, and scaling, together with strong robustness and fault tolerance. Additionally, the learning features have better performance in description of internal information of data and expressiveness [18]. Based on the aforementioned advantages, convolutional features are widely used in matching tasks [19,20,21,22,23,24]. In Fischer et al. [19], CNN deep features were compared with standard SIFT descriptors in terms of region matching and turned out to be superior to SIFT under several typical challenges. In Zagoruyko et al. [20], several CNN-based models were built for comparing image patches, which contain two-channel-based ones, two-stream multi-resolution models, and SPP-based (Spatial-Pyramid-Pooling) Siamese networks. The models can significantly outperform the state-of-art on several benchmark datasets. In Han et al. [21], three fully-connected layers with ReLU (Rectified Linear Units) nonlinearity were used to compute the similarity between the extracted features. In Simo-Serra et al. [22], patch-level correspondence was realized by training deep convolutional models for the extraction of image descriptors. Zbontar et al. [23] addressed the matching cost problem by learning a similarity measure on small image patches using CNN. Combining with the post-processing steps, dense stereo matching can be achieved. Furthermore, Luo et al. [24] replaced the concatenation layer and subsequent processing layers by a single product layer, which shows better performance on efficiency than the works in [23]. Different from the models in [19,20,21,22,23,24], a new keypoint matching method is presented in this paper based on the local multi-layer CNN features, termed Local Convolutional Features (LCFs), which can extract more discriminative features better than the conventional CNNs.

In summary, our main contributions include:

1. A keypoint description method based on CNN deep features is proposed to extract discriminative features.
2. A multi-layer features fusion scheme is exploited to further boost the discrimination of features.
3. A location constraint method is deployed to refine the matching performance of neighboring keypoints.

The rest of this paper is organized as follows. Section 2 summarizes feature extraction based on CNNs. Section 3 illustrates the proposed method for keypoint matching based on LCFs. Section 4 introduces our proposed ice thickness calculation by 3D measurement. Section 5 presents the experimental results and discussion. In addition, Section 6 states the conclusions and future work.

## 2. Feature Extraction Based on Convolutional Neural Networks

A typical CNN model is mainly composed of convolution layer and pooling layer alternately. The convolution layers are used to extract local features, which not only enhance the feature information, but also reduce the noise of input image. While the pooling layers are designed to scale the mapping and reduce the number of parameters, as a result, the extracted features have certain invariance on translation, rotation, and scaling. 

Assume there are *L* layers in a CNN model, and *k* feature maps in the *l*th layer, where *l* = 1, 2, ……, *L*. At a certain layer, the previous layer’s feature maps are convolved with the learning filter kernel $w_{nk}^{l}$ and put through the activation function *F*(·) to output the feature maps $C_{k}^{l}$ [25]. Thus, the *k*th feature map of the *l*th layer can be computed as:

(1) $$C_{k}^{l}=F(\sum_{n\in I_{k}}w_{nk}^{l}*M_{n}^{l-1}+b_{k}^{l})$$

where $M_{n}^{l-1}$ is *n*th feature map in (*l* − *1*)th layer, * represents the convolutional operation, $b_{k}^{l}$ is an additive bias of *n*th feature map in *l*th layer, *I_k_* denotes all the input convolved images of the *k*th feature map. In addition, the activation function *F*(·) is generally a nonlinear activation function which operates component wisely, e.g., the *tanh* or *sigmoid* function. The feature map is generated by accumulating the convolutional multiple input maps. 

The pooling layer enhances the scaling invariance by reducing the spatial resolution of the network, and the output map can be expressed as:

(2) $$S_{k}^{l}=F(\delta_{k}^{l}down(M_{k}^{l-1})+b_{k}^{l})$$

where *down*(·) denotes the down-sampling function, $\delta_{k}^{l}$ is the multiplicative bias, and $b_{k}^{l}$ represents an additive bias. The value of $\delta_{k}^{l}$ varies with the down-sampling method, which usually includes max and mean pooling. The max pooling is used for extracting texture features, while the mean pooling helps to keep the image background. Take the mean pooling as an example, assume $\delta_{k}^{l}$ = 1/*m*, it indicates that each *m* × *m* pixel block is down-sampled and the size of output maps is 1/*m* of the input.

Generally, multi-layer and feed-forward CNN trained with the back-propagation algorithm is widely used to solve the classification problems. For a multiclass problem with *C* classes and *N* training samples, the sample error is represented as:

(3) $$E^{N}=\frac{1}{2}\sum_{n=1}^{N}\sum_{k=1}^{C}{\left(y_{k}^{n}-l_{k}^{n}\right)}^{2}$$

where $y_{k}^{n}$ denotes the *k*th output unit, and $l_{k}^{n}$ is the label of *k*th dimension of the *n*th sample. 

## 3. Proposed Approach

**Figure 1:**
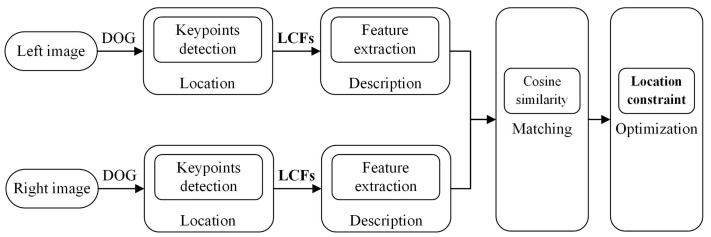
Keypoint matching approach framework. Among them, DOG = difference-of-Gaussian, LCFs = Local Convolutional Features.

As shown in Figure 1, our proposed approach framework for keypoint matching is illustrated. Given the stereo image pairs, we first detect the keypoints as the fundamental elements for matching. Then, we describe the keypoints by the proposed local convolutional features. Next, the features are matched by cosine similarity. Finally, a location constraint method is constructed to optimize the initial matching.

### 3.1. Keypoints Detection

To extract features and build the optimized matching model, the first step is to detect keypoints from the input image. In this paper, the keypoints detection method is based on the widely used difference-of-Gaussian (DOG) method [15], which has advantages of scale invariance and being anti-fuzzy. DOG operator is a method of gray image enhancement and corner detection, which mainly includes scale space construction, local extreme detection, and elimination of poor keypoints. To detect stable keypoint location in scale space more efficiently, the difference-of-Gaussian function is proposed, which can be computed from the difference of two nearby scales. To detect the extreme point in the scale space and two-dimensional image space, the grey value of the target point should be compared with that of the neighboring 26 points. Then, DOG identifies the location and scale accurately by fitting the three-dimensional quadratic function. In addition, the low-contrast and unstable edge response points are removed, which can enhance the matching stability and improve the anti-noise ability.

In Section 5.3.1, some experiments are carried out on PTLI images to compare detection performance between DOG and three classic algorithms, including Hessian, Harris, and FAST.

### 3.2. Local Convolutional Features

The feature description of the keypoint plays a critical role in the matching process. In contrast to the traditional hand-crafted local features, this paper proposes a feature description method by extracting the LCFs.

#### 3.2.1. Keypoints Description Using CNN Features

As mentioned in Section 1, some related CNN-based approaches [19,20,21,22,23,24] have suggested that the CNN-based features outperform the traditional hand-crafted features. To extract the robust and internal features, in this section, we introduce a novel multi-scale feature description method based on local convolutional features. The proposed method allows the full-size image to input the CNN model, and all the features of the keypoints can be extracted once.

Our method is based on the well-known fully convolutional network (FCN) [26] model. In FCN, the fully connected layers are transformed into convolution layers one by one, which makes the input image unconstrained by the fixed size. Moreover, small stride of each layer contributes to keeping details of information. Based on the aforementioned factors, the model FCN-VGG16 (fully convolutional network based on VGG 16-layer net) is utilized to extract convolutional features, and its feature network architecture is shown in Table 1. The focus of this work is on extracting the discriminate features in a new way. 

After the keypoint detection by DOG, we obtain *P* feature points of the input image. Suppose $p_{i}$ is the *i*th keypoints of the image, where $p_{I}$ ∈ *P*. As the analysis in Section 2, the convolutional feature of the keypoint $p_{i}$ in *l*th layer can be written as:

(4) $$V_{i}^{l}=(C_{i1}^{l},C_{i2}^{l},\cdots \cdots ,C_{ik}^{l},\cdots \cdots ,C_{i(N-1)}^{l},C_{iN}^{l})$$

where $C_{ik}^{l}$ is a feature component of the feature map $C_{k}^{l}$ in *l*th layer. It can be seen that $V_{i}^{l}$ is composed of these feature components in order. Intuitively, the feature component $C_{ik}^{l}$ of *k*th feature map in *l*th layer is selected as the feature description of keypoint $p_{i}$. However, it is just one number, which does not provide a rich description. Thus, we extract the feature components of all the feature maps in *l*th layer. In addition, the collection of the feature components is called a local convolutional feature vector $V_{i}^{l}$, which gives a rich description. Apparently, it is critical to find the mapping location of keypoint in each layer.

**Figure 2:**
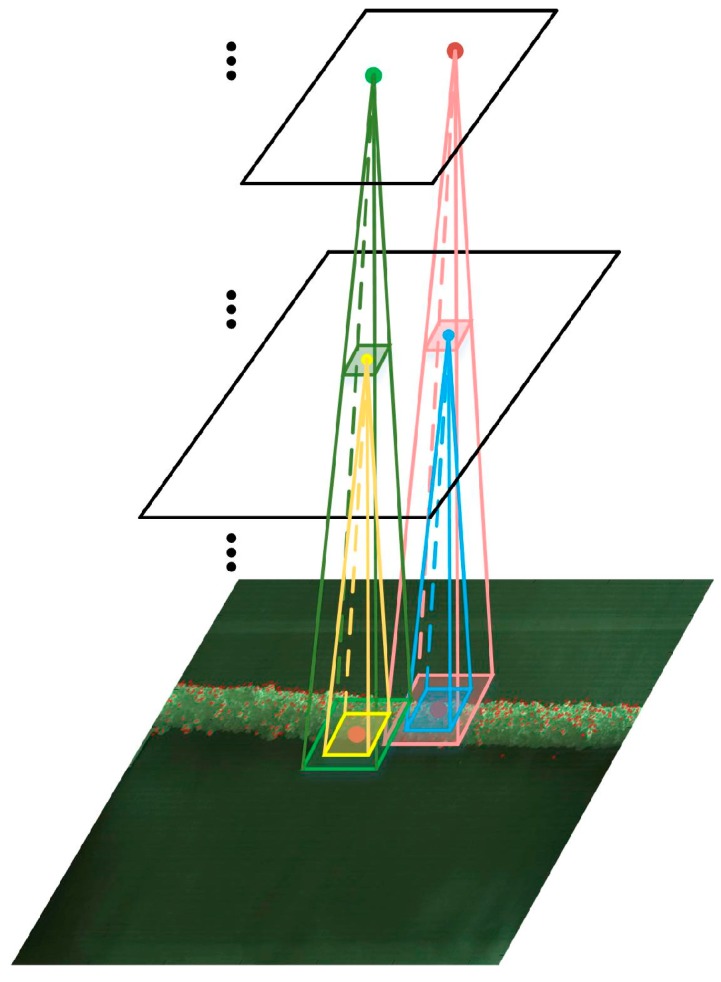
Illustration of corresponding mapping location of keypoint in different layers.

Locations in various layers correspond to the locations in the image they are path-connected to, which are called their receptive fields. Figure 2 illustrates the corresponding mapping location of keypoint in different layers.

Assume the location of keypoint $p_{i}$ in original image is ($x_{i}$, $y_{i}$), the mapping location ($x_{li}$, $y_{li}$) in *l*th layer can be expressed as:

(5) $$\left\{ \begin{array}{l} x_{li}=\frac{x_{i}}{\prod_{s=1}^{S}m_{s}}\\ y_{li}=\frac{y_{i}}{\prod_{s=1}^{S}m_{s}} \end{array}\right.$$

where *s* denotes the number of pooling layers before the *l*th layer, and $m_{s}$ is scale of the *s*th pooling layer. In FCN-VGG16, each convolutional layer does not change the size of input map for that the value of stride is 1. Thus, the mapping location in each layer is mainly influenced by the pooling layers. 

#### 3.2.2. Multi-Layer Features Fusion

In a multi-layer CNN model, the expression abilities of various layers’ features are different, which can be summarized as follows. With the small size of receptive field and shallow convolutional operation, the low-level features can get more accurate location with fewer points matched. In addition, the middle-level features can match larger number of points for the large size of receptive field, however, the matching accuracy is slightly lower than that of low-level features, while the high-level features are not suitable for keypoint matching due to its large receptive field. Thus, a fusion scheme of the low- and middle-level features is proposed to extract robust features, which inherits the advantages of the original features and achieves a better matching performance. Multi-scale information is introduced into the aggregated features, which can boost the discrimination and robust of features.

**Figure 3:**
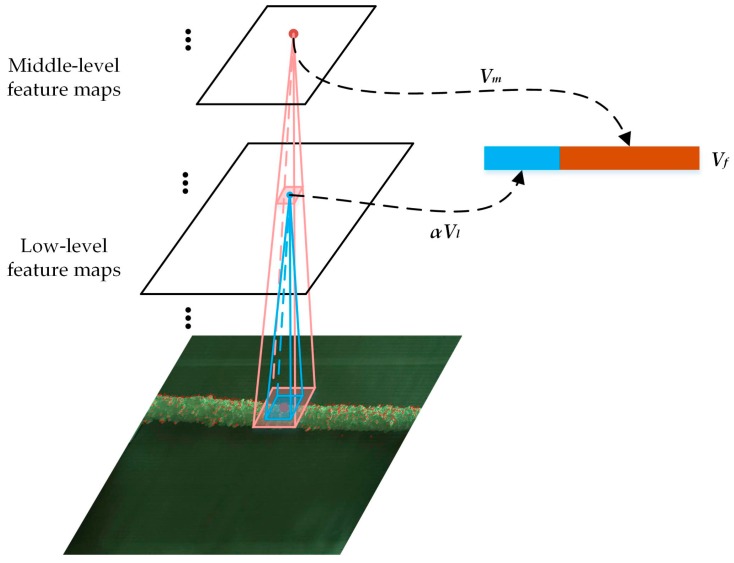
Illustration of multi-layer features fusion.

The visualization of our multi-layer features fusion scheme is shown in Figure 3. After extracting the features in each layer, the aggregated features are formulated as the fusion of features from the low level and middle level layers. In the process of fusion, the initial weight of each layer’s feature is based on its proportion of correct matches, then fine-tuned for the best matching results. The multi-layer features fusion is given as:

(6) $$V_{f}=\{\alpha V_{l},\;V_{m}\}$$

where *α* is a scaling factor. $V_{l}$ and $V_{m}$ are the features from low-level and middle-level layers respectively. Intuitively, $V_{l}$ and $V_{m}$ are concatenated to a single feature vector $V_{f}$, the length of which is the sum of the lengths of $V_{l}$ and $V_{m}$.

### 3.3. Feature Matching

Through the keypoint detection and description, each keypoint is given a multi-dimensional feature. Then, features are matched by cosine similarity for its high running speed and relatively accuracy. Additionally, a distance ratio described in [15,27] is defined for better matching, which is the ratio between the distances of a keypoint from the left image to its nearest keypoint and to its second-nearest keypoint in the right image. The ratio is given as:

(7) $$distratio=\frac{d_{nearest}}{d_{sec-nearest}}$$

where $d_{nearest}$ is the nearest distance and $d_{sec-nearest}$ is the second-nearest distance. We accept the match if the *distratio* is smaller than 0.6, which is set empirically by experiments. The smaller the value of *distratio* is, the fewer points will be matched and higher matching accuracy will be. On the contrary, the number of matched points will increase while the accuracy decreases when the value of *distratio* becomes larger. 

### 3.4. Matching Optimization Based on Location Constraint

Based on the feature extraction of LCFs, an extracted feature corresponds to a region of pixels in the original image. For example, a feature in the conv3_2 layer corresponds to a 4 × 4 region in the original image due to the two pooling operations. Thus, the pixels in this region are described by the same feature. When there are multiple keypoints in this region, the description cannot distinguish them. 

To address this problem, a method for matching optimization based on location constraint is presented, which can use the location information to discriminate each keypoint in the same region. Assume there are $n_{l}$ and $n_{r}$ keypoints correspond to feature $V_{l}$ and $V_{r}$, respectively. If feature $V_{l}$ and $V_{r}$ are matched, the matching relationship of keypoints from these two point sets can be expressed as:

(8) $$\left\{ \begin{array}{cc} P_{l}(\frac{\sum_{i}^{n_{l}}x_{li}}{n_{l}},\frac{\sum_{i}^{n_{l}}y_{li}}{n_{l}})⟷P_{r}(\frac{\sum_{j}^{n_{r}}x_{rj}}{n_{r}},\frac{\sum_{j}^{n_{r}}y_{rj}}{n_{r}}), & n_{l}\neq n_{r}\\ P_{l}(x_{li},y_{li})\overset{cmp(x_{li},x_{rj})}{\underset{cmp(y_{li},y_{rj})}{↔}}P_{r}(x_{rj},y_{rj}), & n_{l}=n_{r} \end{array}\right.$$

where $P_{l}$($x_{li}$, $y_{li}$) is the position of *i*th keypoint in left image and $P_{r}$($x_{rj}$, $y_{rj}$) is that of *j*th keypoint in right image. *cmp*(·) denotes a function that compares the value of the inputting data. When $n_{l}$ ≠ $n_{r}$, the central position of $n_{l}$ points corresponds to that of $n_{r}$ points. When $n_{l}$ = $n_{r}$, the correspondence of each point from the two point sets is determined by comparing the location information. After a series of experiments using DOG operator, it was found that the values of $n_{l}$ and $n_{r}$ are not lager than 3 in a 4 × 4 region, furthermore, the probability of $n_{l}$ = 3 ($n_{r}$ = 3) is small.

## 4. Ice Thickness Calculation Using 3D Measurement

**Figure 4:**
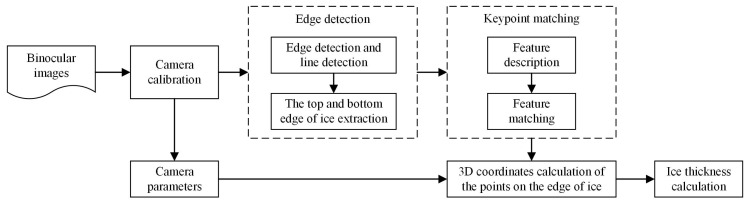
The flowchart of ice thickness measurement.

Ice thickness is the key indicator of ice disaster. In this section, we present an approach to ice thickness measurement based on the proposed keypoint matching method. Figure 4 illustrates the flowchart of the ice thickness calculation. Given the binocular images, we first compute the camera parameters through camera calibration. Then, edge detection is used to find the top and bottom boundary of ice formed on power transmission lines. Next, the 3D coordinates of the keypoints on the edge of ice are computed by using the proposed keypoint matching method. Finally, the ice thickness is calculated. 

Assume *T* = ($p_{1}^{t}$, $p_{2}^{t}$, $p_{3}^{t}$,… $p_{i}^{t}$… $p_{\left(n-1\right)}^{t}$, $p_{n}^{t}$) and *B* = ($p_{1}^{b}$, $p_{2}^{b}$, $p_{3}^{b}$,… $p_{j}^{b}$… $p_{\left(m-1\right)}^{b}$, $p_{m}^{b}$) are keypoints near the top and bottom of ice edges, respectively. The 3D coordinates of those keypoints are obtained through the 3D measurement process. Thus, the top line $L_{t}$ and bottom line $L_{b}$ of ice edge can be fitted easily. The distance *D* between these two lines can be considered as the total thickness after icing. Apparently, *D* is the sum of wire diameter and twice the ice thickness. If the diameter of the power line is $d_{l}$, then the ice thickness can be expressed as:

(9) $$d=\frac{D-d_{l}}{2}$$

## 5. Experiments and Evaluation

In this section, we evaluate the proposed method on two different challenging tasks: the actual collected simulated PTLI scene dataset and Middlebury dataset. On the PTLI dataset, we verify the effectiveness of the proposed method on the ice thickness measurement. In addition, on the Middlebury dataset, the overall performance of our method is evaluated in terms of matching precision and recall. A keypoint pair is considered to be a correct match if the error of true match is within 3 pixels. Moreover, the repetitive matches are deleted for reasonable comparison.

### 5.1. Datasets and Evaluation Metrics

#### 5.1.1. Datasets

The matching performance of our proposed method is evaluated on the actual collected simulated PTLI scene dataset and Middlebury stereo dataset. On the one hand, the image pairs of simulated PTLI dataset are collected via Daheng binocular camera. It is a huge and difficult work doing pixel-level labeling on collected images, thus, the measurement results of ice thickness by the proposed method are directly compared with the results of manual measurement. On the other hand, Middlebury is a stereo dataset, with each part published in five different works in the years 2001, 2003, 2005, 2006, 2014, respectively [28,29,30,31,32]. The image pairs of this database are indoor scenes taken controlled lighting conditions, and the density and precision of true disparities are high via using structured light measurement. The dataset is divided into 35 training sets and 25 testing sets, and the resolution of image pairs is selected the smallest–size of the given configuration. There are ten thousand keypoints, which are enough for point matching test.

#### 5.1.2. Evaluation Metrics

Two evaluation metrics described in [33] are adopted to assess the matching performance. The first criterion is precision, which is based on the number of correct matches (*# correct matches*) with respect to the number of all matched points (*# all matches*) by the matching algorithm. The formula is shown as:

(10) $$precision=\frac{\#correct\;matches}{\#all\;matches}$$

The second criterion is recall, namely the number of correct matches with respect to the number of corresponding points (*# true matches*) between input image pairs. It can be expressed as:

(11) $$recall=\frac{\#correct\;matches}{\#true\;matches}$$

### 5.2. Performance of the Proposed Method

#### 5.2.1. Experiments Using Various Layers’ Features

**Figure 5:**
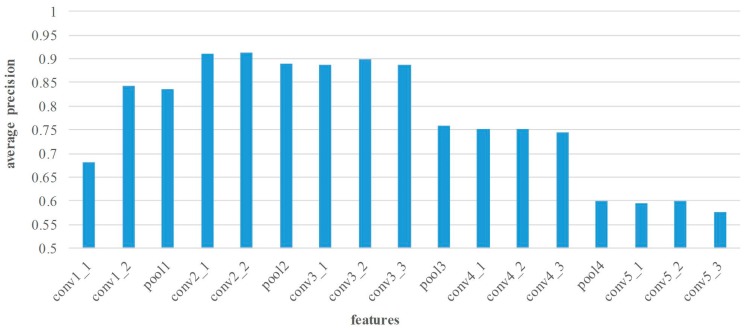
Average matching precision comparison of various layers’ features on Middlebury dataset. The horizontal axis denotes the various layers’ features, and the vertical axis is the average matching precision.

**Figure 6:**
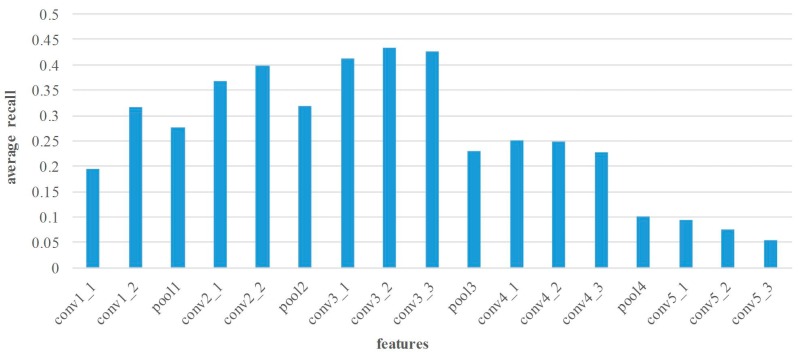
Average matching recall comparison of various layers’ features on Middlebury dataset. The horizontal axis denotes the various layers’ features, and the vertical axis is the average matching recall.

In this section, the matching performance of various layers’ features is tested at all image pairs from Middlebury dataset. Thus, the features from different layers are extracted by the proposed method. The statistical results of precision and recall are shown in Figure 5 and Figure 6.

Figure 5 illustrates the average matching precision of various layers’ features. In general, the features of pooling layers show lower performance. As the number of layers continuously increase, the matching precision of convolutional layers may increase before decreasing. For the small receptive field and few convolutions, the matching performance of layer conv1 is poor. On the contrary, the features of layer conv2 and conv3 are suitable for point matching because of the appropriate receptive field and convolutional operation. Apparently, the conv2_2 features achieve the superior performance in matching precision, and the average precision is 91.3%. Nevertheless, too large receptive field leads to lower performance of the high layer features, such as layer conv4 and conv5 features. Figure 6 shows the average matching recall of various layers’ features. The distribution of recall is similar to that of precision, and the highest recall 42.9% can be achieved by using conv3_2 features.

#### 5.2.2. Importance of the Proposed Feature Fusion scheme and Location Constraint Method

**Figure 7:**
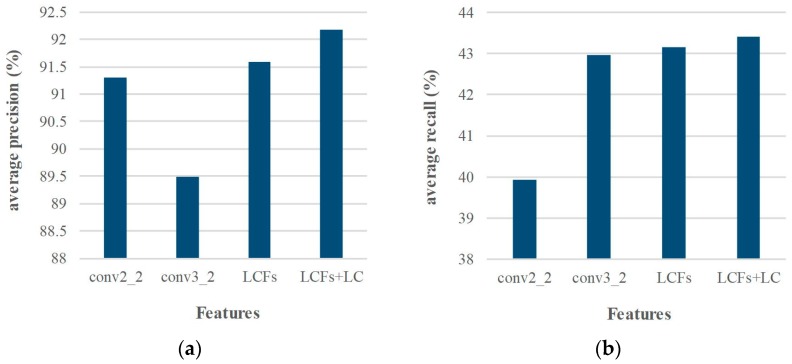
The matching performance using different features. Among them, LC = Location Constraint. (**a**) average precision comparison; (**b**) average recall comparison.

To prove the effect of the proposed multi-layer features fusion scheme and location constraint method in matching performance, we also evaluate it on Middlebury dataset. The LCFs features are the integration of conv2_2 and conv3_2 features, and the weight parameter *α* is chosen as 0.6 based on experimental validations. From the experimental results of various layers’ features, conv2_2 and conv3_2 features are the best candidates in terms of matching precision and recall, respectively. Figure 7 gives the matching comparison of LCFs with the conv2_2 and conv3_2 features. The matching precision of LCFs 91.6% is higher than conv2_2 features 91.3% and conv3_2 features 89.5%, and the recall of LCFs 43.2% is also higher than conv2_2 features 39.9% and conv3_2 features 42.9%. It is shown that the feature fusion scheme has already achieved promising performance. Furthermore, with the proposed location constraint optimization, the matching precision goes up from 91.6% to 92.2%, recall from 43.2% to 43.5%. It indicates that our optimization method has improved the matching performance without affecting the efficiency. In addition, we note that the optimization method mainly works when the keypoints fall in the same region. In summary, our method achieves excellent results in terms of precision and recall. Furthermore, the proposed idea is also flexible enough to combine any layer features to achieve further performance boost.

### 5.3. Ice Thickness Measurement

In this section, experiments are carried out on ice thickness measurement by using the proposed keypoint matching method at the actual collected simulated PTLI scene dataset. 

#### 5.3.1. Keypoint Detection with Different Operators

**Figure 8:**
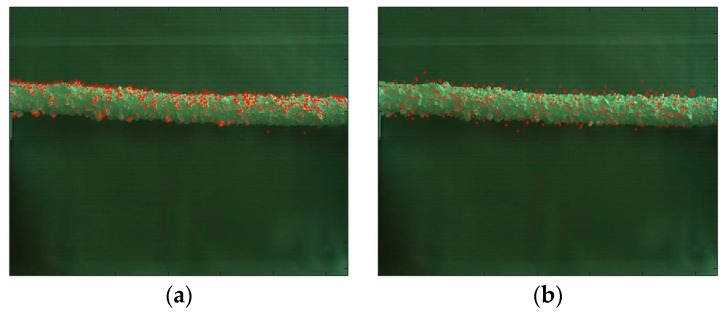

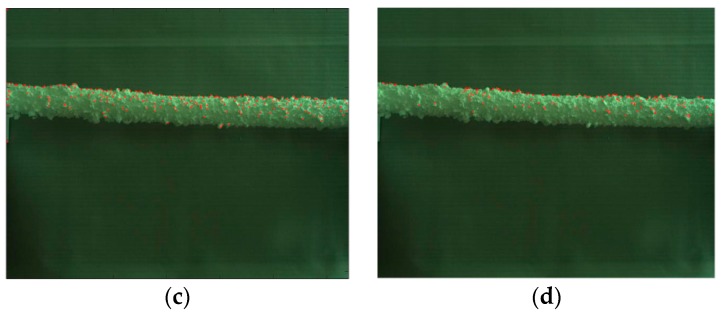
Keypoint detection examples on the collected dataset. (**a**) DOG; (**b**) Hessian; (**c**) Harris; (**d**) FAST.

Keypoint detection is the basis for feature extraction and matching. Figure 8 shows the detection results by DOG, Hessian, Harris and FAST in the PLTI scene. It presents that: (1) by contrast, the number of detected keypoints of DOG is the largest, and enough edge-points provide a guarantee for ice thickness measurement. (2) Hessian is less sensitive to illumination, and its detection speed is faster than DOG while the extraction of edge-points is not much. (3) the standard Harris does not have invariance in terms of scale transformation. (4) FAST runs the fastest; however, its robustness is poor when the noise is high, and the detection results depend on the value of the threshold t. Based on the analysis above, this paper utilizes DOG to detect keypoints.

#### 5.3.2. Results of Ice Thickness Measurement

With 3D measurement principle of binocular stereo vision, the 3D information of the keypoint is determined by camera parameters and the correspondence of the keypoint. Through Zhang Zhengyou calibration method [34], the stereo calibration results of our binocular stereo camera are as follows.

(12) $$KK\_left=\left[\begin{array}{ccc} 2405.78292 & 0 & 542.17752\\ 0 & 2429.85134 & 394.52050\\ 0 & 0 & 1 \end{array}\right],\;KK\_right=\left[\begin{array}{ccc} 2528.76308 & 0 & 447.60667\\ 0 & 2522.2384 & 449.83808\\ 0 & 0 & 1 \end{array}\right]$$

(13) $$R=\left[\begin{array}{lll} 0.9995846 & 0.0096263 & -0.0271654\\ -0.0111616 & 0.9983151 & -0.0569422\\ 0.0265715 & 0.0572218 & 0.9980078 \end{array}\right],\;T=\left[\begin{array}{l} 172.68493\\ 32.17323\\ 57.83972 \end{array}\right]$$

Here, *KK_left* and *KK_right* are the internal parameters of left and right camera. *R* and *T* are the rotation matrix and the translation matrix between the coordinate system.

In this experiment, ten image pairs of PTLI scene are used for testing the measurement accuracy of ice thickness. The ice thickness values of specified locations from the ten image pairs are obtained by our method and manual measurement respectively. In addition, the manual measurement results are measured by Vernier caliper with the accuracy of 0.02 mm. Table 2 shows the comparison results in detail. It shows that the absolute error is acceptable for ice thickness measurement. Assume the true value of ice thickness is the manual measured value, the average accuracy of our method can reach 90.9%.

### 5.4. Benchmark Comparisons

#### 5.4.1. Comparison with Other Feature Description Methods

In this part, the proposed method LCFs is compared with four state-of-the-art algorithms at 25 test image pairs from Middlebury dataset, including traditional hand-crafted features SIFT, SURF, ORB as well as CNN-based models MatchNet [21]. MatchNet is a typical method for comparing image patches in recent years. For fair comparison, we modify the matching process of all approaches under the same metric shown in Section 3.3. In addition, the mismatched points are not removed in all methods. Moreover, the repetitive matches are deleted for reasonable comparison. In the experiment of MatchNet, we use the pre-trained model to compute the matching scores of the patches (with the size of 64 × 64). In addition, the patches are considered as a matching pair if their output score is 1. 

**Figure 9:**
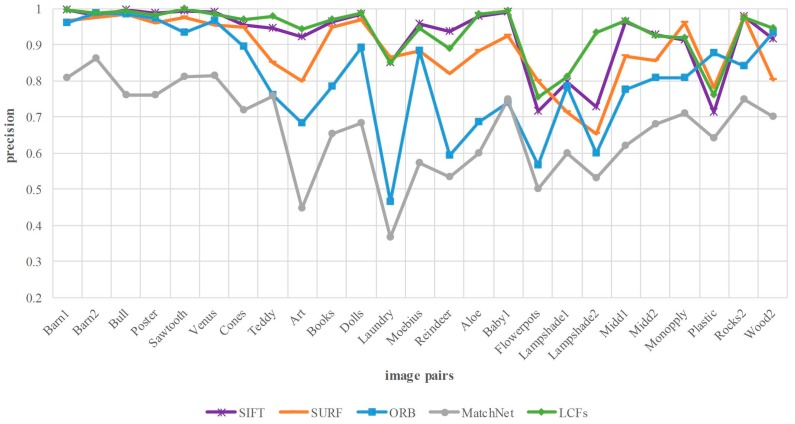
Matching precision comparison between our method and four comparing approaches on Middlebury dataset. The horizontal axis denotes the image pairs, and the vertical axis is the matching precision.

Figure 9 demonstrates that LCFs is better than SURF, ORB in most cases except for a few, besides, it achieves comparable performance with SIFT in terms of matching precision. Our approach achieves 1.5%, 5.3%, 13.1%, 27.3% improvement in the average precision compared with SIFT, SURF, ORB and MatchNet. MatchNet can only output the matching score of two image patches, which makes it inefficient to implement the matching between the two images without other optimization matching strategies. Furthermore, there are patches that match with multiple patches due to the low discrimination of the model without being fine-tuned. 

**Figure 10:**
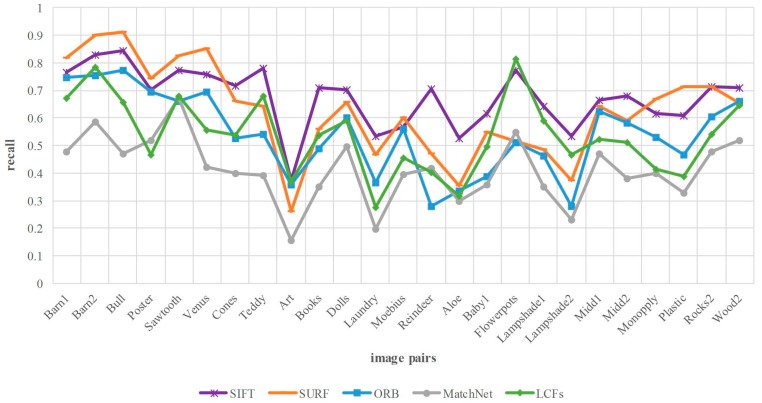
Matching recall comparison between our method and four comparing approaches on Middlebury dataset. The horizontal axis denotes the image pairs, and the vertical axis is the matching recall.

Figure 10 reports the comparison of matching recall. Above all, the number of matches depends on the keypoints detection method and matching approach. The detection operator of our method is DOG, the same with SIFT, while the operators of SURF and ORB are Hessian and FAST, respectively. The results show that the proposed method is lower than SIFT in terms of matching recall. As we know, the correspondence of the keypoints depends on whether the corresponding features are matched. However, for the case of high similarity and dimension, cosine similarity treats each feature component equally and does not take interrelation into account. As a result, some correct matches are discarded in the process of matching. This problem can be addressed by integrating more discriminative metrics into our framework, which help to verify the correspondence between the similar features. This topic will be investigated in our future research.

#### 5.4.2. Comparison with Outlier Removal Method 

After the process of the proposed method, the initial correspondences have been established. The initial correspondence typically includes most of the true matches, but still a small number of false matches. The purpose of our method is to get more accurate initial matching. It is known that keypoint matching can be further improved by false matching removal. In this section, we conduct comparative experiments to examine the effect of outlier removal. The method [14] is used to remove the false matches of the initial matching. 

Table 3 shows the experimental results in detail. By combining with method [14], the final average precision of SIFT goes up from 92.3% to 98.1%. As for LCFs, the final average precision is improved from 93.8% to 99.6%. The work of outlier removal can effectively improve the final matching accuracy. Moreover, it also indicates that our method gets a more stable initial match than SIFT dose. In terms of recall, the average recall is slightly reduced after the outlier removal process. This is because some correct matches are deleted while the outlier is removed. Therefore, we will focus on the outlier removal algorithms in the future work.

## 6. Conclusions

This paper proposes a new keypoint matching method based on the local convolutional features. First, a novel keypoint description method that utilizes the CNN features is proposed, which can extract local discriminative features. Then, a fusion scheme of multi-layer features is presented to aggregate the low and middle level CNN features. Since combining the multi-scale information, LCFs can extract more discriminative features. In addition, a location constraint method is exploited to refine the correspondence of neighboring keypoints. We apply the proposed method to measure the ice thickness in PTLI scene, and the average accuracy can reach 90.9% compared with manual measurement. Finally, the experimental results show that the proposed approach achieves 1.5%, 5.3%, 13.1%, 27.3% improvement in the average precision compared with SIFT, SURF, ORB and MatchNet on Middlebury dataset. In the future, we aim to use metric learning and outlier removal algorithm to optimize the matching performance. Furthermore, we will apply the proposed method to Unmanned Aerial Vehicle (UAV) power line inspection [35,36], and combine our method with correlation filter [37] for the other applications.

## Figures and Tables

**Table 1:** Feature network architecture of fully convolutional network based on VGG 16-layer net (FCN-VGG16) (C: convolution, MP: max-pooling).

Name	Type	Output Dim.	Kernel Size	Stride
conv1_1	C	64	3 × 3	1
conv1_2	C	64	3 × 3	1
pool1	MP	64	2 × 2	2
conv2_1	C	128	3 × 3	1
conv2_2	C	128	3 × 3	1
pool2	MP	128	2 × 2	2
conv3_1	C	256	3 × 3	1
conv3_2	C	256	3 × 3	1
conv3_3	C	256	3 × 3	1
pool3	MP	256	2 × 2	2
conv4_1	C	512	3 × 3	1
conv4_2	C	512	3 × 3	1
conv4_3	C	512	3 × 3	1
pool4	MP	512	2 × 2	2
conv5_1	C	512	3 × 3	1
conv5_2	C	512	3 × 3	1
conv5_3	C	512	3 × 3	1
pool5	MP	512	2 × 2	2

**Table 2:** Experimental results of ice thickness (mm).

Number	1	2	3	4	5	6	7	8	9	10
Manual measurement	7.12	10.10	13.98	8.92	11.85	10.60	5.85	10.50	8.34	9.80
LCFs	6.83	11.26	12.85	8.60	10.98	11.32	7.46	11.31	9.26	9.49
Absolute error	0.29	1.16	1.13	0.32	0.87	0.72	1.61	0.81	0.92	0.31

**Table 3:** Experimental results with outlier removal method.

Method	Precision (Average)	Recall (Average)
SIFT	92.3%	67.4%
LCFs	93.8%	53.6%
SIFT + [14]	98.1%	66.9%
LCFs + [14]	99.6%	53.1%

## References

[1] Jiang X., Xiang Z., Zhang Z., Hu J., Hu Q., Shu L. (2014). Predictive model for equivalent ice thickness load on overhead transmission lines based on measured insulator string deviations. IEEE Trans. Power Deliv..

[2] Ma G.M., Li C.R., Quan J.T., Jiang J., Cheng Y.C. (2011). A fiber bragg grating tension and tilt sensor applied to icing monitoring on overhead transmission lines. IEEE Trans. Power Deliv..

[3] Zarnani A., Musilek P., Shi X., Ke X., He H., Greiner R. (2012). Learning to predict ice accretion on electric power lines. Eng. Appl. Artif. Intell..

[4] Farzaneh M., Savadjiev K. (2005). Statistical analysis of field data for precipitation icing accretion on overhead power lines. IEEE Trans. Power Del..

[5] Lu J.Z., Zhang H.X., Fang Z., Li B. (2009). Application of self-adaptive segmental threshold to ice thickness identification. High Volt. Eng..

[6] Gu I.Y.H., Berlijn S., Gutman I., Bollen M.H.J. Practical applications of automatic image analysis for overhead lines. Proceedings of the 22nd International Conference and Exhibition on Electricity Distribution (CIRED 2013).

[7] Hao Y., Liu G., Xue Y., Zhu J., Shi Z., Li L. (2014). Wavelet image recognition of ice thickness on transmission lines. High Volt. Eng..

[8] Yu C., Peng Q., Wachal R., Wang P. An Image-Based 3D Acquisition of Ice Accretions on Power Transmission Lines. Proceedings of the CCECE’06 Canadian Conference on Electrical and Computer Engineering.

[9] Wachal R., Stoezel J., Peckover M., Godkin D. A computer vision early-warning ice detection system for the Smart Grid. Proceedings of the Transmission and Distribution Conference and Exposition.

[10] Yang H., Wu W. (2012). On-line monitoring method of icing transmission lines based on 3D reconstruction. Autom. Electr. Power Syst..

[11] Ma J., Zhao J., Yuille A L. (2016). Non-rigid point set registration by preserving global and local structures. IEEE Trans. Image Process..

[12] Ma J., Zhou H., Zhao J., Gao Y., Jiang J., Tian J. (2015). Robust feature matching for remote sensing image registration via locally linear transforming. IEEE Trans. Geosci. Remote Sens..

[13] Ma J., Qiu W., Zhao J., Ma Y., Yuile A.L., Tu Z. (2015). Robust L2E Estimation of Transformation for Non-Rigid Registration. IEEE Trans. Signal Process..

[14] Ma J., Zhao J., Tian J., Yuile A.L., Tu Z. (2014). Robust point matching via vector field consensus. IEEE Trans. Image Process..

[15] Lowe D.G. (2004). Distinctive Image Features from Scale-Invariant Keypoints. Int. J. Comput. Vis..

[16] Bay H., Tuytelaars T., Van Gool L. SURF: Speeded Up Robust Features. Proceedings of the 9th European Conference on Computer Vision.

[17] Rublee E., Rabaud V., Konolige K., Bradski G. ORB: An efficient alternative to SIFT or SURF. Proceedings of the IEEE International Conference on Computer Vision.

[18] Zhang B., Yang Y., Chen C., Yang L., Han J., Shao L. (2017). Action recognition using 3D histograms of texture and a multi-class boosting classifier. IEEE Trans. Image Process..

[19] Fischer P., Dosovitskiy A., Brox T. (2014). Descriptor matching with convolutional neural networks: a comparison to sift. arXiv.

[20] Zagoruyko S., Komodakis N. (2015). Learning to compare image patches via convolutional neural networks. Comput. Vis. Pattern Recogn..

[21] Han X., Leung T., Jia Y., Sukthankar R., Berg A.C. MatchNet: Unifying feature and metric learning for patch-based matching. Proceedings of the IEEE Conference on Computer Vision and Pattern Recognition.

[22] Simo-Serra E., Trulls E., Ferraz L., Kokkinos I., Fua P., Morenonoguer F. Discriminative Learning of Deep Convolutional Feature Point Descriptors. Proceedings of the International Conference on Computer Vision.

[23] Zbontar J., LeCun Y. (2016). Stereo matching by training a convolutional neural network to compare image patches. J. Mach. Learn. Res..

[24] Luo W., Schwing A.G., Urtasun R. Efficient Deep Learning for Stereo Matching. Proceedings of the IEEE Conference on Computer Vision and Pattern Recognition.

[25] Wang L., Zhang B., Han J., Shen L., Qian C.S. (2016). Robust object representation by boosting-like deep learning architecture. Signal Process. Image Commun..

[26] Long J., Shelhamer E., Darrell T. Fully convolutional networks for semantic segmentation. Proceedings of the IEEE Conference on Computer Vision and Pattern Recognition.

[27] Li D., Zhou H., Lam K.M. (2015). High-resolution face verification using pore-scale facial features. IEEE Trans. Image Process..

[28] Scharstein D., Szeliski R. (2002). A taxonomy and evaluation of dense two-frame stereo correspondence algorithms. Int. J. Comput. Vis..

[29] Scharstein D., Szeliski R. High-Accuracy Stereo Depth Maps Using Structured Light. Proceedings of the IEEE Computer Society Conference on Computer Vision and Pattern Recognition.

[30] Scharstein D., Pal C. Learning Conditional Random Fields for Stereo. Proceedings of the IEEE Conference on Computer Vision and Pattern Recognition.

[31] Hirschmuller H., Scharstein D. Evaluation of Cost Functions for Stereo Matching. Proceedings of the IEEE Conference on Computer Vision and Pattern Recognition.

[32] Scharstein D., Hirschmüller H., Kitajima Y., Krathwohl G., Nešić N., Wang X., Westling P., Jiang X., Hornegger J., Koch R. (2014). High-Resolution Stereo Datasets with Subpixel-Accurate Ground Truth. Pattern Recognition.

[33] Mikolajczyk K., Schmid C. (2005). A performance evaluation of local descriptors. IEEE Trans. Pattern Anal. Mach. Intell..

[34] Zhang Z. (2000). A flexible new technique for camera calibration. IEEE Trans. Pattern Anal. Mach. Intell..

[35] Li Z., Liu Y., Walker R., Hayward R., Zhang J. (2010). Towards automatic power line detection for a UAV surveillance system using pulse coupled neural filter and an improved Hough transform. Mach. Vis. Appl..

[36] Zhang B., Liu W., Mao Z., Liu J., Shen L. (2014). Cooperative and geometric learning algorithm (CGLA) for path planning of UAVs with limited information. Automatica.

[37] Zhang B., Luan S., Chen C., Han J., Wang W., Perina A., Shao L. (2018). Latent Constrained Correlation Filter. IEEE Trans. Image Process..

